# Biginelli Reaction Catalyzed by Copper Nanoparticles

**DOI:** 10.1371/journal.pone.0043078

**Published:** 2012-08-17

**Authors:** Manika Dewan, Ajeet Kumar, Amit Saxena, Arnab De, Subho Mozumdar

**Affiliations:** 1 Department of Chemistry, University of Delhi, Delhi, India; 2 Department of Microbiology and Immunology, Columbia University Medical Center, New York, New York, United States of America; Queen’s University Belfast, United Kingdom

## Abstract

We recently reported a novel synthesis of copper nanoparticles from copper sulphate utilizing the charge-compensatory effect of ionic liquid [bmim]BF_4_ and ethylene glycol. The nanoparticles were characterized and found to be stable for one year. Here we hypothesize that the stabilized nanoparticles should be able to catalyze one-pot multicomponent organic reactions. We show that the nanoparticles catalyzed Biginelli reaction at room temperature to give the product 3,4-dihydopyrimidinone (>90% yield in ∼15 minutes) from aldehydes, β-diketoester (ethylacetoacetate) and urea (or thiourea). ). Remarkably, such high yields and rapid kinetics was found to be independent of the electronic density on the reactant aryl-aldehyde. This was probably because even the surface-active particles reacted faster in the presence of ionic liquid as compared to conventional methods. The heterocyclic dihydropyrimidinones (DHPMs) and their derivatives are widely used in natural and synthetic organic chemistry due to their wide spectrum of biological and therapeutic properties (resulting from their antibacterial, antiviral, antitumor and anti-inflammatory activities. Our method has an easy work-up procedure and the nanoparticles could be recycled with minimal loss of efficiency.

## Introduction

The world today is experiencing the benefits of metal nanoparticles in a host of different areas including but not limited to optics, electronics and medicine [1]. Copper is an example of a metal nanoparticle which has gained considerable attention in the past two decades due to its unusual properties, leading to potential applications in many diverse fields. To exemplify, non-agglomerated, spherical, uniform copper nanoparticles finds use in lubrication, as nanofluids and catalysts, etc [2], [3]. Hence not surprisingly, a number of methods such as microemulsion, reverse micelles, gamma irradiation, UV light irradiation, protecting electrolytic techniques by controlling electrode potential and the polyol process have been developed for the preparation of copper nanoparticles [4]. A one phase system using alkanethiolate as a protecting monolayer has been described for the synthesis of copper nanoparticles [5]. Besides, sonochemical methods and thermal decomposition methods have also been reported [6], [7]. However, the copper nanoparticles synthesized by these methods have their limitations as they have a limited size, are monodispersed and susceptible to oxidation. Hence, there is a need to develop a methodology to synthesize copper nanoparticles with increased stability. We postulated that ionic liquids could be used in this respect to confer the stability to the nanoparticles.

Ionic liquids (ILs) have already emerged as a green alternative to the conventional and environmentally detrimental volatile solvents [8]. They have attracted a great deal of attention due to their high thermal stability, good conductivity, non volatility, non flammability, suitable polarity, wide electrochemical window and recyclability [8]–[11]. Most importantly, the physical and chemical properties of ILs can be exploited by altering their cation, anion and attached substituents [12], [13]. The aforementioned properties of ILs have been used extensively and they continue to be potentially useful for use in sensors [14], material synthesis [15]–[17], separation and extraction [18], asymmetric synthesis [19], nuclear fuel cycle processing [20], liquid thermal storage media and heat transfer fluids [21], lubricants [22], etc.

Imidazolium based ionic liquids like [bmim][BF_4_] has been selected as novel reaction media for promoting various organic transformation reactions because of its high miscibility with water [23]. It has been shown that [bmim][BF_4_] can increase the rate of diazocoupling between 4-substituted benzenediazonium tetrafluoroborates and β-naphthol in the presence of triethylamine [24]. Because of its hydrophilicity, convenient viscosity and ease of handling, [bmim][BF_4_] has also been used for synthesis of N-arylphthalimides (an important class of imide derivative substrates for biological and chemical applications) [25]. Imidazolium ILs are liquids at room temperature and provide an excellent medium for the formation and stabilization of transition metal nanoparticles. Their negligible vapor pressure allows the size and shape of the metal nanoparticles to be investigated *in situ* by TEM [26], [27]. Typically, particles synthesized in organic solvents are immiscible with water and this severely limits their applicability. Many applications require that the nanoparticles be dispersed and stable in water. However, water based synthesis of nanoparticles is fraught with many problems such as ionic interactions, low reactant concentration, and difficulty in removing the stabilizers [28]. Ionic liquids could be used to overcome this as both the cation and anion of an ionic liquid can potentially serve as charge compensating groups in the synthetic procedure. When an ionic liquid is used as a reaction media, the solute is solvated by ions only. Thus, the reaction can proceed in a completely different environment as compared to when water or organic solvents are used. As a result, high selectivity is possible [29]. Our study provides an alternative to synthesizing nanomaterials with minimal energy consumption and high yield. We have previously synthesized and isolated well dispersed and size controlled copper nanoparticles in a ionic liquid *[bmim]BF_4_* - ethylene glycol system without the aid of any heating or microwave irradiation [30]. These nanoparticles were found to be highly stable for one year.

**Figure 1:**
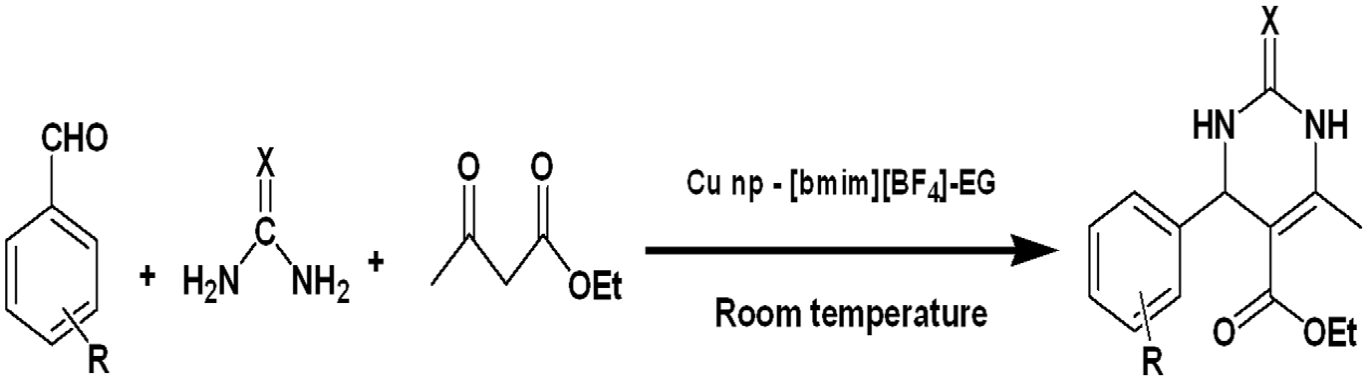
Synthesis of 3,4 dihydropyrimidin-2-ones at room temperature.

**Figure 2:**
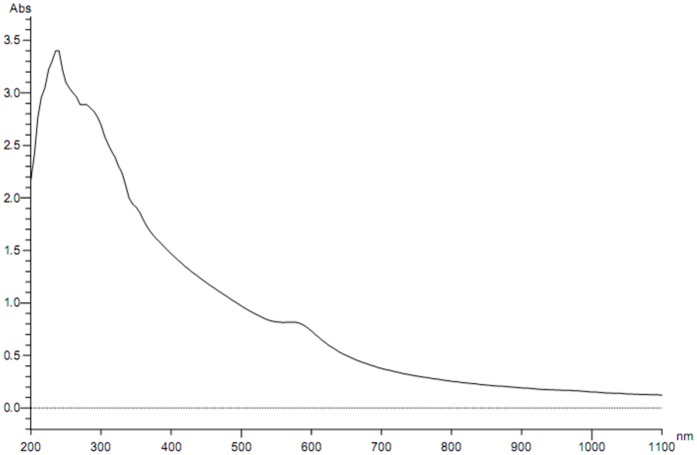
UV-Visible spectra of Copper nanoparticles formed in “[bmim]BF_4_ - ethylene glycol” system.

**Figure 3:**
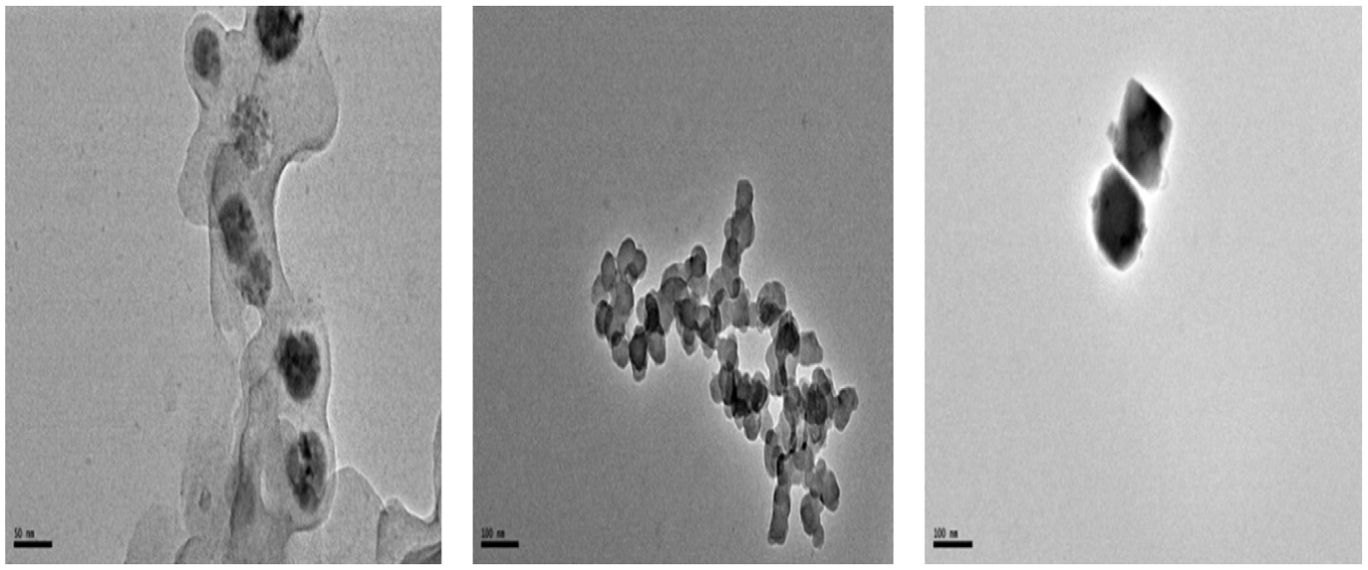
TEM images of Copper nanoparticles formed in “[bmim]BF_4_ - ethylene glycol” system.

**Figure 4:**
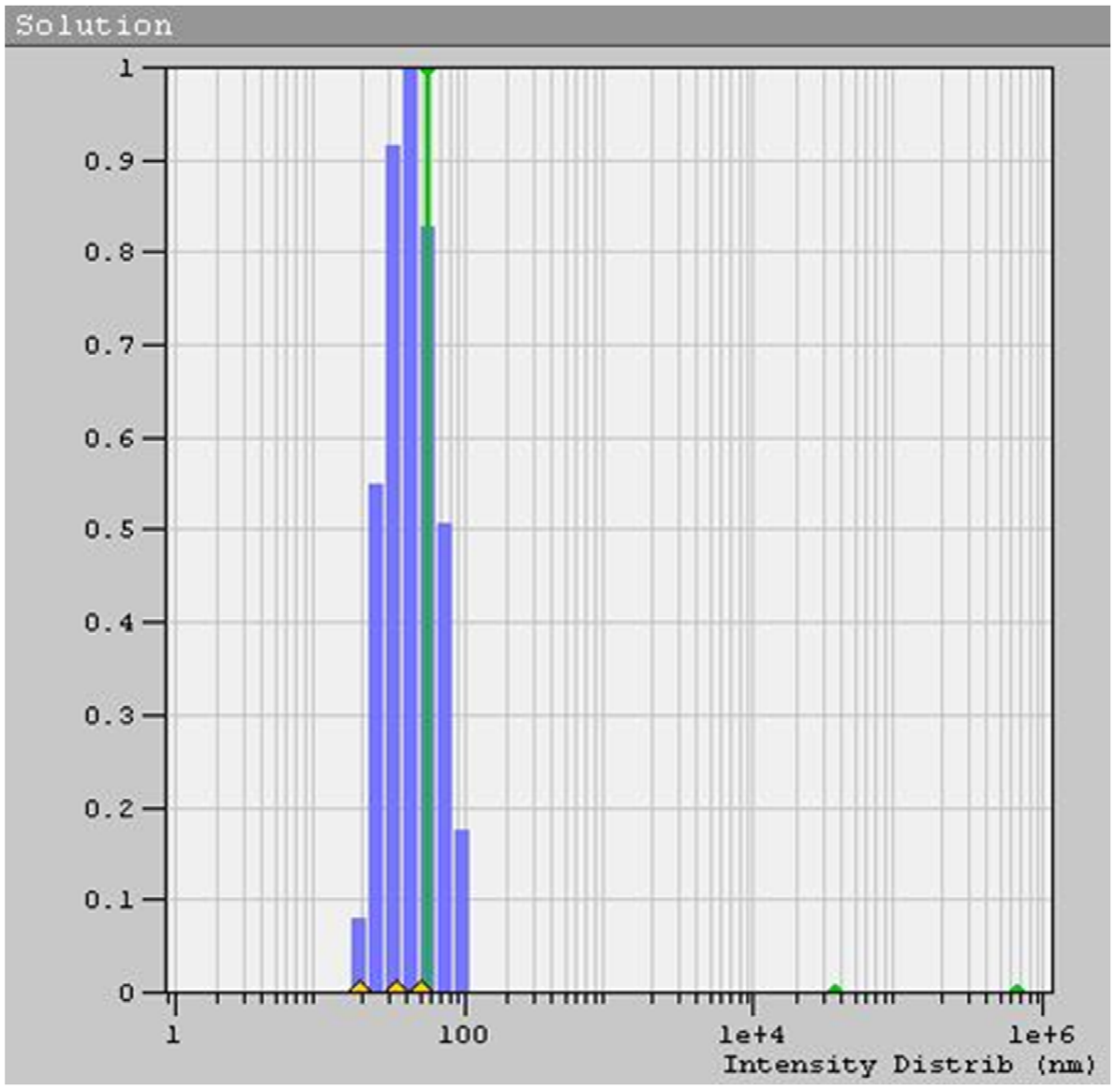
Dynamic Light Scattering data of copper nanoparticles as prepared by this method “[bmim]BF_4_ - ethylene glycol” system.

**Figure 5:**
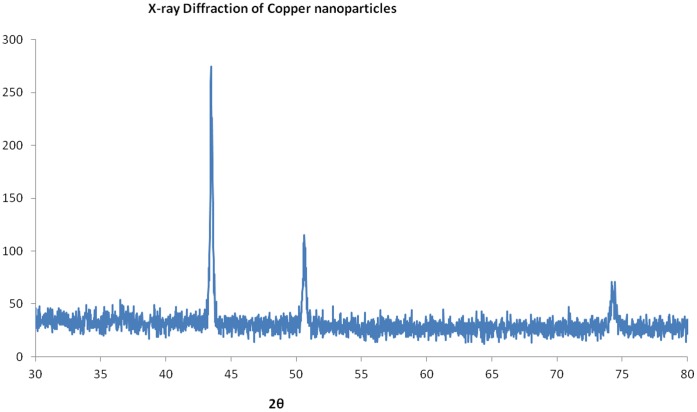
XRD plot of Copper nanoparticles formed in “[bmim]BF_4_ - ethylene glycol” system.

We hypothesize that the stabilized nanoparticles should be able to catalyze one-pot multicomponent organic reactions. Hence, the synthesized copper nanoparticles were tested as catalysts for Biginelli reaction and the product 3,4-dihydropyrimidinones (DHPMS) was formed in the presence of the ionic liquid [bmim]BF_4_ as a solvent. Dihydropyrimidinones (DHPMs) and their derivatives are heterocyclic units and widely used in natural and synthetic organic chemistry due to their wide spectrum of biological and therapeutic properties (resulting from their antibacterial, antiviral, antitumor and anti-inflammatory activities) [31]. Recently, appropriately functionalized DHPM analogs have emerged as integral backbones of several calcium channel blockers, antihypertensive agents and α-la-adrenergic receptor antagonists [32]. Moreover, several alkaloids containing the dihydropyrimidine core unit have been isolated from marine sources and these have displayed interesting biological properties. In particular, the batzelladine alkaloids have been found to be potent HIV gp-120-CD4 inhibitors [33]. In view of the versatile properties of dihydropyrimidinones, the development of a novel, cost effective synthetic strategy is of immense importance. The reaction conditions traditionally employed involve strong Bronsted and Lewis acids, such as LiClO4, LaCl3–7H2O, InCl3, Bi(OTf)3, BiCl3, Mn(OAc)3, Cu(OTf)2, FeCl3–6H2O, ZrCl4 or SnCl2.2H2O [34]. Among the Si-MCM-41 or montomorillonite K 10 clay supported ZnCl2, AlCl3, GaCl3, InCl3 and FeCl3 catalysts, FeCl3/Si-MCM-41 has shown best results for microwave assisted synthesis of dihydropyrimidinones [35]. Formic acid has been used under solventless conditions for microwave irradiated synthesis of these compounds [36] while Cu(OTf)2 has also been used at 100°C in the presence of ethanol [37]. There have also been previous reports on the use of solid-phase protocols which allow higher degree of throughput and automation [38]. An earlier method to synthesize dihydropyrimidinones in ionic liquid involved heating and this was the possible reason why the catalyst could not be recycled [39]. Additionally, the yields obtained with some of the aldehydes is low. Similarly, dihydropyrimidinones have been synthesized using Cu(BF4)2. xH2O as a catalyst; however as in the previous case, the catalyst has recyclability issues [40]. Metal nanoparticles has been previously demonstrated to efficiently catalyze a variety of organic reactions [41]–[47]. Here we have used copper nanoparticles dispersed in ionic liquid/ethylene glycol for the synthesis of 3,4dihydropyrimidinones at room temperature with reduced reaction times and much higher yields (Supporting information S1, graphical abstract; Figure 1). The nanoparticles retained its efficacy after multiple cycles of reaction.

**Figure 6:**
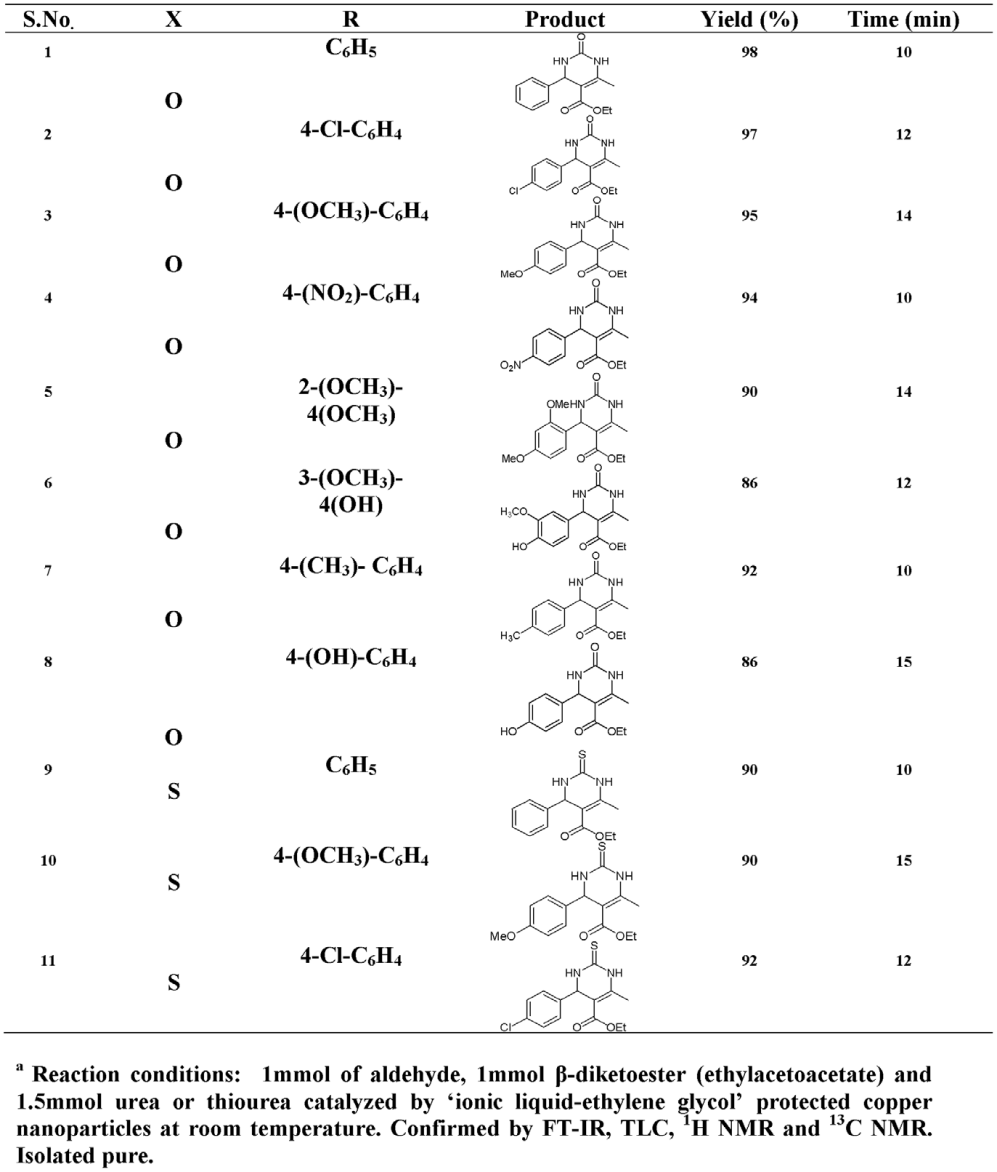
Biginelli reaction catalyzed by ‘Ionic liquid- ethylene glycol’ protected copper nanoparticles at room temperature^a^.

## Experimental

### 1. Materials

Copper sulphate pentahydrate (CuSO_4_.5H_2_O), ethylene glycol, sodium tetrafluoroborate and 1-butyl-3-methylimidazoliumbromide were all of analytical grade and used as such. All the aldehydes and solvents were purchased from spectrochem Pvt. Ltd. Mumbai (India) and were used without any additional purification. All reactions were monitored by thin layer chromatography (TLC) on gel F254 plates. ^1^H-NMR and ^13^ C-NMR spectra were recorded in CDCl_3_ and DMSO-d_6_ on a Jeol JNN ECX- 400P spectrometer; Melting points were recorded on SECOR Laboratories instruments melting point instruments. The infrared spectra were recorded using a model Perkin Elmer spectrum BX2 FT-IR system. Spectra were recorded with Spectrum V 5.3.1 software in the range 4000–400 cm^−1^. The KBr pellet technique was adopted for recording the spectra.

### 2. Synthesis of Ionic Liquid (IL) [bmim]BF_4_ and Preparation of Copper Nanoparticles in IL-Ethylene Glycol Media

Sodium tetrafluoroborate and 1-butyl-3-methylimidazoliumbromide in equimolar quantities were stirred in dry acetone under anhydrous conditions for 48 h. The mixture was filtered off to remove unreacted sodium tetrafluoroborate and the filtrate was further treated with dichloromethane to remove sodium bromide and again the filtrate obtained was again treated with dichloromethane to check for any further precipitation. The solvents were removed under reduced pressure and the resulting colorless ionic liquid was dried in rotavapor at 70°C for 2 h to remove water. The product 1-butyl-3- methylimidazoliumtetrafluoroboratewas characterized by ^1^H NMR studies as reported by us previously. Subsequently copper nanoparticles was prepared as described in the presence of ethylene glycol and ionic liquid *[bmim]BF_4_* (24). An aliquot amount of ‘ionic liquid-ethylene glycol’ protected copper nanoparticles were taken out and particle size distribution measurements were done using particle size analyzer (QELS, Photocor-FC, model-1135 P). Transmission electron microscope (TEM, FEI Technai 300 kV fitted with EDAX) was used to image size and morphology of the powder. X-ray diffraction patterns of the powders were recorded using diffractometer (Philips Analytica PW 1830 X-ray equipped with a 2θ compensting slits).

### 3. General Procedure for Synthesis of 3,4-dihydropyrimidinones

A mixture of aldehyde (1 mmol), β-diketoester (ethylacetoacetate) (1 mmol) and urea (or thiourea) (1.5 mmol) was stirred for fifteen minutes in the presence of 100 µl of highly dispersed reaction mixture containing ‘ionic liquid-ethylene glycol’ protected copper nanoparticles from the previous experiment (Figure 1). The reaction was monitored by thin layer chromatography using ethyl acetate/hexane (2∶3) as eluent. The mixture was then extracted thrice with 10 ml of ethyl acetate, drying the organic layer in vacuum afforded 3,4-dihydropyrimidinones.The ionic liquid residue was washed with hexane and dried in vacuum resulting in a recycled catalytic system which could be used over three turns.

**Table 1:** Effect of various catalysts employed in Biginelli reaction catalyzed by **‘**Ionic liquid- ethylene glycol’ protected copper nanoparticles at room temperature^a^.

S.No	Reaction conditions	Time	Yield
1	Without catalyst	No reaction	No reaction
2	[bmim]BF_4_	No reaction	No reaction
3	Bare Cu nanoparticles	Oxidation of nanoparticle	No reaction
4	Cu nanoparticles dispersed in ethylene glycol	Oxidation of nanoparticles after 5 min	No reaction
5	Cu nanoparticles stabilized with [bmim]BF_4_–ethylene glycol	10 min	98%
6	Hydrazine hydrate	Sticky mass; incomplete reaction.	

a Reaction conditions: 1 mmole benzaldehyde, 1 mmole ethylacetoacetate and 1.5 mmole urea catalysed by ‘ionic liquid-ethylene glycol’ protected copper nanoparticles at room temperature.

**Table 2:** Reuse of catalytic system containing copper nanoparticles stabilised by [bmim]BF_4_-ethylene glycol for Biginelli reaction^a^.

Run	Table I, Entry 1		Table I, Entry 7	
	Time (min)	Yield (%)^b^	Time (min)	Yield (%)^b^
1	10	98	10	90
2	10	86	10	89
3	10	80	10	77

a Reaction condition: 1 mmole aldehyde, 1 mmole ethylacetoacetate, 1.5 mmole urea catalyzed by ‘ionic liquid-ethylene glycol’ protected copper nanoparticles at room temperature.

b Confirmed by FT-IR, TLC, ^1^H NMR and ^13^C NMR. Isolated pure.

**Figure 7:**
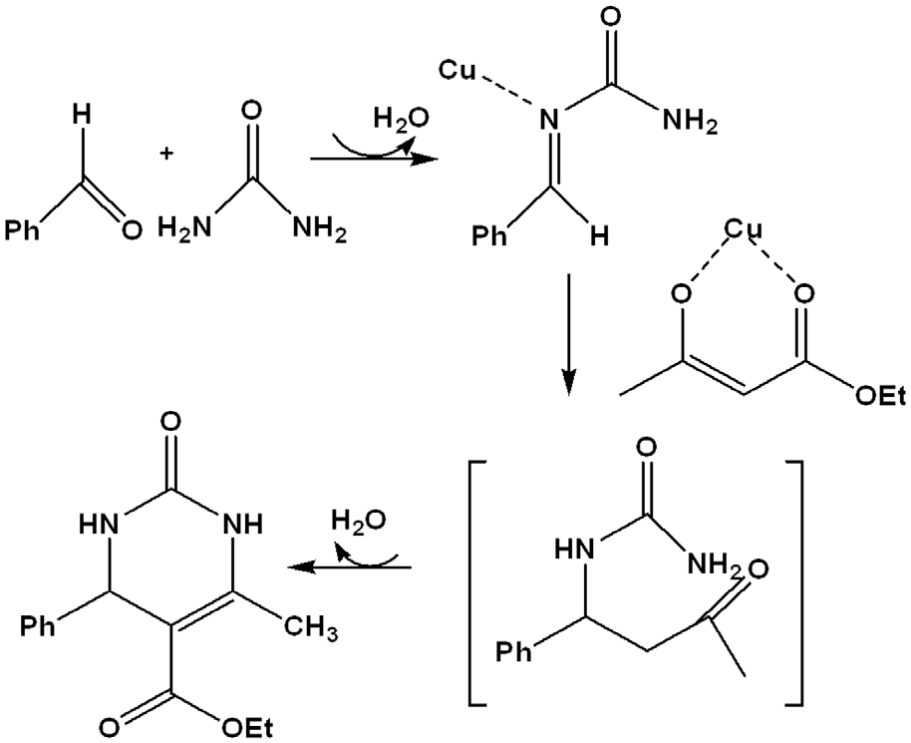
Proposed mechanism for the synthesis of 3,4-dihydropyrimidin-2-ones using ‘Ionic liquid- ethylene glycol’ protected copper nanoparticles.

## Results and Discussion

We discover that ionic liquid in conjunction with ethylene glycol play a vital role in the stabilization of copper nanoparticles, and both the ionic liquid and ethylene glycol play an important role in the process. The ionic liquid [bmim]BF_4_, serves as an excellent media for dispersing copper nanoparticles, controlling their size and preventing their aerial oxidation; however agglomeration could not be avoided in the absence of ethylene glycol. On the other hand, while the reaction takes place effectively in the absence of hydrophilic ionic liquid [bmim]BF_4_, the stability of the formed particles could not be extended for longer periods of time.

After separating the copper nanoparticles from the suspension, their shape and size distribution was analyzed by DLS and TEM. The average sizes were calculated from the XRD peak data. The UV-Visible spectra of Copper nanoparticles formed in “[bmim]BF_4_ - ethylene glycol” system is shown in Fig. 2. TEM images in Fig. 3 illustrates the formation of spherical, ‘ionic liquid-ethylene glycol’ protected copper nanoparticles with little agglomeration. Fig. 4 shows the size distribution from DLS data, revealing a mean size of 40±3 nm in diameter with polydispersity index of 0.203. XRD patterns of the copper nanoparticles prepared from this procedure was plotted in Fig. 5, and it has 2θ values from 20° to 90°. XRD pattern reflections correspond to that of pure copper, showing three peaks corresponding to indices: (111), (200) and (220); which is the characteristic of pure copper. Thus, these peaks accurately resemble oxide-free FCC copper phase with no impurity.

The Biginelli reaction involving benzaldehyde (and other variously substituted aryl-aldehydes) (1 mmol), ethylacetoacetate (1 mmol) and urea or thiourea (1.5 mmol) was successfully catalyzed by copper nanoparticles dispersed in ionic liquid [bmim]BF_4_ at room temperature. The results of the reaction are represented in Figure 6. The yield of the product and the time taken for the reaction is reported alongside indicating the efficacy of the catalyst at room temperature. A control reaction was carried out to test the requirement of a catalyst by stirring the reagents in the absence of the catalyst. No desirable products could be detected in this case.

Furthermore, it was shown that ionic liquid alone was ineffective in catalyzing the model reaction (Figure 1) at room temperature in the absence of copper nanoparticles. Interestingly, the copper nanoparticles were also unable to catalyze the reaction by itself as they were inadvertently oxidized in the absence of the stabilizing ionic liquid. (Table 1). Moreover, the copper nanoparticles dispersed in ethylene-glycol (but in the absence of ionic liquid [bmim]BF_4_) could also not catalyze the reaction as the unprotected nanoparticles are oxidized within 5 minutes. This is consistent with our previous observation that both ethylene glycol and ionic liquid [bmim]BF_4_ is required for synthesizing stable, monodispersed nanoparticles. This also demonstrated that stabilized copper nanoparticles are required for catalyzing the Biginelli reaction. The stable catalytic system could be reused successively for three turns (Table 2) with negligible loss of activity. ‘Ionic liquid-ethylene glycol’ protected copper nanoparticles retained 80% of their efficacy after being recycled three times as evidenced by the reactions of compounds 1 and 7 in Figure 6 (with C_6_H_5_ and 4-(CH_3_)- C_6_H_4_ substituents respectively) to yield the corresponding 3,4-dihydropyrimidinones (Table 2).

The proposed mechanism in Figure 7 illustrates the postulated interaction of the acylimine intermediate with copper nanoparticles. A cursory examination of the reaction mechanism suggests that electron withdrawing groups in the aryl aldehyde would accelerate the rate of the reaction. However, it is intriguing to note that the reaction proceeded rapidly to afford such high yields irrespective of whether electron-withdrawing or electron- donating side chains was attached to the reactant aryl aldehyde. This could be attributed to the surface-active particles reacting faster in the presence of ionic liquid as compared to conventional methods. Taken together, these results prove the efficacy of the catalytic system (copper nanoparticles stabilized by ionic liquid- ethylene glycol mixture) as a catalyst for the synthesis of biologically active 3,4-dihydropyrimidinones.

### Conclusion

The results laid down by these experiments proved the efficacy of the copper nanoparticles stabilized by ionic liquid- ethylene glycol mixture as catalysts for the synthesis of biologically active 3,4-dihydropyrimidinones. The products were obtained rapidly with high yields at room temperature.

## Supporting Information

Supporting Information S1 **Graphical Abstract.**
(DOC)
