# Supplementary material for: Biginelli Reaction Catalyzed by Copper Nanoparticles
Source: PLoS One. 2012 Aug 17;7(8):e43078. doi: 10.1371/journal.pone.0043078 (PMC3422326; doi:10.1371/journal.pone.0043078)
Supplement: Supporting Information S1 — Graphical Abstract. (DOC) [file pone.0043078.s001.doc]

**GRAPHICAL ABSTRACT**

Biginelli reaction catalyzed by copper nanoparticles

Manika Dewan, ^a^ Ajeet Kumar,^a^ Amit Saxena,^a^ Arnab De,^b^ Subho Mozumdar^a^

^a^Department of Chemistry, University of Delhi, Delhi-110007, India

^b^Department of Microbiology and Immunology, Columbia University Medical Center, New York, USA

E-mail: [subhoscom@yahoo.co.in](mailto:subhoscom@yahoo.co.in)
